# The First-In-Class Anti-AXL×CD3ε Pronectin™-Based Bispecific T-Cell Engager Is Active in Preclinical Models of Human Soft Tissue and Bone Sarcomas

**DOI:** 10.3390/cancers15061647

**Published:** 2023-03-08

**Authors:** Nicoletta Polerà, Antonia Mancuso, Caterina Riillo, Daniele Caracciolo, Stefania Signorelli, Katia Grillone, Serena Ascrizzi, Craig A. Hokanson, Francesco Conforti, Nicoletta Staropoli, Luigia Gervasi, Maria Teresa Di Martino, Mariamena Arbitrio, Giuseppe Nisticò, Roberto Crea, Pierosandro Tagliaferri, Giada Juli, Pierfrancesco Tassone

**Affiliations:** 1 Department of Experimental and Clinical Medicine, Magna Græcia University, 88100 Catanzaro, Italy; 2 Protelica, Inc., Hayward, CA 94545, USA; 3 Pathology Unit, Annunziata Hospital, 87100 Cosenza, Italy; 4 Institute of Research and Biomedical Innovation (IRIB), Italian National Council (CNR), 88100 Catanzaro, Italy; 5 Renato Dulbecco Institute, 88046 Lamezia Terme, Italy; 6 College of Science and Technology, Temple University, Philadelphia, PA 19122, USA

**Keywords:** sarcomas, AXL, pronectins™, bispecific T-cell engager, BTCE, immunotherapy, cancer

## Abstract

**Simple Summary:**

Sarcomas are a group of heterogeneous diseases with a poor prognosis and scarce therapeutic options. Innovative approaches based on novel therapeutic targets are eagerly awaited. AXL, a TAM family tyrosine kinase receptor, recently emerged as an interesting target for several type of sarcomas. Here, we propose an innovative immunotherapeutic strategy based on the targeting of AXL, using a first-in-class Pronectin™-based Bispecific T-Cell Engager (pAXL×CD3ε) for the treatment of sarcomas. Our results demonstrate that pAXL×CD3ε redirects T cells toward AXL-expressing sarcoma cell lines, leading a dose-dependent and T cell-mediated cytotoxicity in vitro. Moreover, pAXL×CD3ε inhibits the in vivo growth of human sarcoma xenografts and improves survival in immunocompromised mice, thus representing a new-generation strategy for the treatment of a still-incurable disease.

**Abstract:**

Sarcomas are heterogeneous malignancies with limited therapeutic options and a poor prognosis. We developed an innovative immunotherapeutic agent, a first-in-class Pronectin™-based Bispecific T-Cell Engager (pAXL×CD3ε), for the targeting of AXL, a TAM family tyrosine kinase receptor highly expressed in sarcomas. AXL expression was first analyzed by flow cytometry, qRT-PCR, and Western blot on a panel of sarcoma cell lines. The T-cell-mediated pAXL×CD3ε cytotoxicity against sarcoma cells was investigated by flow cytometry, luminescence assay, and fluorescent microscopy imaging. The activation and degranulation of T cells induced by pAXL×CD3ε were evaluated by flow cytometry. The antitumor activity induced by pAXL×CD3ε in combination with trabectedin was also investigated. In vivo activity studies of pAXL×CD3ε were performed in immunocompromised mice (NSG), engrafted with human sarcoma cells and reconstituted with human peripheral blood mononuclear cells from healthy donors. Most sarcoma cells showed high expression of AXL. pAXL×CD3ε triggered T-lymphocyte activation and induced dose-dependent T-cell-mediated cytotoxicity. The combination of pAXL×CD3ε with trabectedin increased cytotoxicity. pAXL×CD3ε inhibited the in vivo growth of human sarcoma xenografts, increasing the survival of treated mice. Our data demonstrate the antitumor efficacy of pAXL×CD3ε against sarcoma cells, providing a translational framework for the clinical development of pAXL×CD3ε in the treatment of human sarcomas, aggressive and still-incurable malignancies.

## 1. Introduction

Sarcomas are a large group of heterogeneous malignancies of mesenchymal origin, commonly characterized by a poor prognosis, of which the onset may occur at any age [1,2]. Among them, soft tissue sarcomas (STSs) represent 80%, bone sarcomas 15% and gastrointestinal stromal tumors 5%. Because of their heterogeneity and common aggressive nature, they are resistant to available therapies and clinical management is still highly challenging [3,4,5]. Conventional treatment, including surgery, radiation therapy and chemotherapy (Doxorubicin, Ifosfamide, trabectedin, and others), differs from one subtype to another. Surgery is the first-line treatment for localized sarcomas, in combination with pre- or post-operative therapies [6], while chemotherapy is the standard treatment for metastatic disease. Unfortunately, the median survival for advanced disease is around 12 months [7]. In this scenario, targeted therapies which might overcome the limitations of current treatments are eagerly awaited [8]. Different signaling pathways involved in sarcoma genesis have been investigated so far. Targeting therapies involving (i) cell cycle progression, through cell cycle inhibitors (CDKIs) [9,10]; (ii) and growth receptors and pro-survival signaling molecules, through tyrosine kinase inhibitors (TKIs) [11], IGFR [12] and mTOR inhibitors [13], have shown efficacy against sarcomas, but only the VEGR inhibitor pazopanib has reached the prime time [14]. Inhibition of epigenetic regulators [15] and poly-ADP-ribose-polymerase (PARP) inhibitors have also demonstrated promising anti-cancer activity in preclinical and clinical studies [16].

Even if immunotherapy may be considered a new therapeutic path and some clinical trials based on the use of immune checkpoint inhibitors are currently ongoing [17,18], to date, it is not considered a valuable option for most sarcomas. Other clinical trials are investigating strategies based on endogenous, transgenic, or chimeric antigen receptor (CAR)-expressing T cells for the targeting of specific antigens, such as tyrosine-kinase-like orphan receptor 2, CD133, GD-2, Muc1 and CD117 (e.g., NCT03356782, NCT00902044, NCT04995003, NCT01953900) (https://clinicaltrials.gov/, accessed on 10 February 2023) [19]. Nevertheless, the effective treatment of advanced disease is an unmet clinical need, and most sarcomas can still be considered incurable. Novel strategies based on new therapeutic targets are highly desirable.

Recently, the AXL receptor has emerged as a promising candidate target for a variety of sarcomas [20,21,22]. The AXL gene is located on chromosome 19q13.2. It encodes for the protein called AXL (UFO, ARK, Tyro7, or JTK11), a member of the TAM family of tyrosine kinase receptors (RTKs) characterized by an extracellular, transmembrane, and intracellular domain [22]. The extracellular structure consists of two immunoglobulins (Ig-like) and two fibronectin type III (Fro III-like) chains, while the intracellular domain is important for auto-phosphorylation and signaling kinase activity [23]. In normal cells and tissues, AXL regulates cell survival, non-inflammatory clearance of apoptotic cells, natural killer cell differentiation and platelet aggregation. AXL is also expressed in cancer cells and microenvironmental immune cells, including dendritic cells, macrophages, and NK cells. It drives several cellular processes that are critical for the development, growth and spread of tumors, including proliferation, invasiveness and migration, epithelial–mesenchymal transition, angiogenesis, and immune resistance [24,25,26].

Different therapeutic agents targeting AXL have been recently developed, including: (i) small molecule inhibitors, which block AXL auto-phosphorylation and kinase activities, such as BGB324, presently investigated in phase I/II clinical trials [27]; (ii) anti-AXL monoclonal antibodies (mAbs), such as YW327.6S2, which bind both human and murine AXL [22]; (iii) nucleotide aptamers, such as the RNA aptamer GL21.T [28]; (iv) soluble AXL receptor that acts as a decoy receptor for the AXL ligand GAS6; (v) natural compounds, such as the *Viscum album* (L.) extract [29].

It was also demonstrated that AXL is over-expressed in Kaposi sarcoma and Kaposi sarcoma herpesvirus-transformed endothelial cells. MAbs generated to induce AXL degradation inhibited Kaposi-sarcoma-cell invasion in in vitro models and tumor growth in vivo [30]. A subsequent study identified AXL as a potential therapeutic target for Ewing sarcoma. AXL inhibitors were shown to affect the viability of Ewing sarcoma cells [31]. In addition, a high expression of AXL gene was found in leiomyosarcoma, and its activity was suppressed by two different multi-tyrosine kinase inhibitors (Crizotinib and Foretinib) [20]. Finally, other studies have found that osteosarcoma cells highly express AXL [32], of which the inhibition significantly reduces lung metastases [21]. Despite all these promising findings, an effective anti-AXL treatment is still not available for these aggressive malignancies.

A novel class of non-immunoglobulin, single-domain therapeutic proteins (Pronectins™), based on “antibody mimics” technology, has been just developed with the aim of providing a novel platform for the treatment of various diseases, including cancer. Pronectins™ were isolated from synthetic human libraries, built upon the 14th domain of Fibronectin III (14FN3) scaffold, which is selected by a bioinformatic approach, and on advanced complementarity-determining region (CDR) diversity of more than 25 billion loop sequences [33]. Since Pronectins™ mimic the natural human repertoire, they are poorly immunogenic [34]. Several pharmacological properties are associated to the fibronectin III scaffold, such as high stability, tissue penetration, and low cost of production. Furthermore, they are smaller than a conventional mAb, representing a favorable feature for the local delivery to the solid tumor mass [35]. Starting from Pronectins™, it is possible to generate multimers, fusion proteins, bispecifics or constructs with site-specific modifications for tailored therapy [36]. Bispecific T-Cell Engagers (BTCEs) emerged as a novel promising strategy for hematologic malignancies, but their application in solid tumors is highly challenging, due to the paucity of selective tumor-associated antigens (TAAs) and the struggle in penetrating the solid tumor mass [37]. In this scenario, a Pronectin™-based BTCE (pBTCE) can help overcome these limitations.

Based on this rationale, we investigated the in vitro and in vivo activity of a first-in-class pBTCE targeting AXL (pAXL×CD3ε) as a potential immunotherapeutic agent for the treatment of sarcomas.

## 2. Materials and Methods

### 2.1. Generation and Development of pAXL×CD3ε

A highly specific anti-AXL Pronectin™, a non-immunoglobulin and single-domain protein, has been isolated from synthetic libraries based on the human scaffold of the 14th domain of fibronectin III (14FN3), as previously described [38]. By bioinformatic analysis aimed to select the best candidate within the amino acid loop diversity and minimize or prevent immunogenicity, 6 Pronectins™ with a KD < 10 nM were identified and AXL54 was chosen for targeting purposes (KD = 8 nM). This Pronectin™ was used to develop a first-in-class BTCE (AXL54 (Pronectin™)-linker-scFV CD3, pAXL×CD3ε), for investigation as an anti-tumor novel agent. The linker is made of a single unit of Gly4–Ser (GGGGS) [38].

### 2.2. Cell Lines

CAL-72, ESS-I, HT-1080, SAOS-2 and Rh-30 were purchased by DSMZ. SW982 and RD-ES were purchased from ATCC. ESS-I (endometrial stromal sarcoma), SAOS-2 (osteogenic sarcoma), SW982 (synovial sarcoma) and RD-ES (Ewing’s sarcoma) were grown in RPMI 1640 (Gibco^®^, Thermo Fisher Scientific, Waltham, MA, USA), supplemented with 20% fetal bovine serum (FBS) (Lonza Group Ltd., Basel, Switzerland), penicillin (100 U/mL) and streptomycin (100 µg/mL) (Gibco^®^, Thermo Fisher Scientific). Rh-30 (rhabdomyosarcoma) was cultured in RPMI 1640, supplemented with 10% FBS, 100 U/mL penicillin and 100 µg/mL streptomycin. CAL-72 (osteosarcoma) and HT-1080 (fibrosarcoma) cell lines were cultured in DMEM-GlutaMAX™ (Gibco^®^, Thermo Fisher Scientific), respectively, supplemented with 20% FBS and 10% FBS, 100 U/mL penicillin and 100 µg/mL streptomycin. Cell lines were maintained at 37 °C, in a humidified atmosphere with 5% CO_2_.

### 2.3. Transduction of Sarcoma Cell Lines

Sarcoma cells were plated at 1 × 10^5^ cells/mL in 6-well plate and incubated O/N. To obtain sarcoma cells stably expressing green fluorescent protein (GFP) transgene, a lentiviral GFP-encoding vector was added according to the manufacturer’s instruction (SBI System Biosciences, Mountain View, CA, USA). Polybrene (Sigma-Aldrich, Saint Louis, MO, USA) was also used to a final concentration of 8 µg/mL. Two days after transduction, cells were selected using DMEM, supplemented with 20% FBS, containing 1 mg/mL puromycin (Sigma Aldrich). After antibiotic selection, puromycin-resistant transduced cells were assessed for the expression of GFP by flow cytometry, using Attune NxT Flow cytometer (Thermo Fisher Scientific) and microscopy (Thunder Imaging Systems, Leica Microsystems, Wetzlar, Germany).

### 2.4. Peripheral Blood Mononuclear Cell (PBMC) Isolation

Mononuclear cells were obtained from healthy donor buffy coats. Briefly, PBMCs were isolated by Ficoll-Paque Plus (Cytiva Europe GmbH, Buccinasco, Milan, Italy) density gradient centrifugation, according to the manufacturer’s recommendations, and washed twice in the culture medium (RPMI-1640 supplemented with 10% FBS), as previously described [39,40].

### 2.5. Detection of AXL Expression and Target Quantification

AXL expression was analyzed on each sarcoma cell line by flow cytometry. Cells were incubated with FITC-conjugated AXL antibody (#MAB154-100, R&D Systems, Minneapolis, MN, USA) for 15 min at RT in the dark. The tubes were washed in PBS 1X and centrifuged 400× *g* for 5 min, resuspended in 500 µL of PBS 1X and analyzed by a flow cytometer.

To quantify AXL expression on sarcoma cell lines, calibrated microspheres (Quantum Simply Cellular, Bangs Laboratories Inc., Fishers, Castenaso, BO, Italy) were used according to the manufacturer’s protocol. Briefly, saturating amounts of FITC-conjugated AXL antibody were added to one drop of each microbead suspension, and the final mixes were incubated for 30 min at RT in the dark. Samples were washed twice using PBS 1X (2500× *g*), resuspended in 500 µL of PBS 1X and analyzed by a flow cytometer. Simultaneously, each cell line was stained with FITC-conjugated AXL antibody, as previously described. The analysis was performed maintaining the same instrument setting used for QSC beads. A QuickCal^®^ spreadsheet, provided by Bangs Laboratories, was used to convert the main fluorescence intensity (MFI) from microspheres to antibody-binding capacity (ABC) values.

### 2.6. Redirected T-Cell Cytotoxicity Assay

PBMCs were isolated from at least 3 donors and labeled with CellTrace™ Violet viable marker (Invitrogen, Waltham, MA, USA), according to the manufacturer’s instructions, and co-cultured with sarcoma cell lines (CAL-72, ESS-I, HT-1080, SAOS-2, Rh-30, SW982 or RD-ES) at different effector-to-target-cell (E:T) ratio, in the presence of increasing concentrations of pAXL×CD3ε (0.1 µg/mL, 1 µg/mL and 2.5 µg/mL) or anti-B-cell maturation antigen (BCMA) Pronectin™-based BTCE, pBCMA×CD3ε (2.5 µg/mL), as a negative control. BCMA is in fact highly restricted to hematopoietic B cells and is not expressed by solid tumors, therefore representing a suitable negative control in our case. Cells were incubated for 72 h at 37 °C and 5% CO_2_, and finally stained with 7-AAD (BD Biosciences, La Jolla, CA, USA). The cytotoxic effect on sarcoma cell lines was detected by flow cytometry and reported as the percentage of 7-AAD^+^/CellTrace™ Violet^–^ cells. The 10:1 E:T ratio was selected because it allowed for the highest toxicity.

Cells stably expressing GFP gene were co-cultured with PBMCs from at least 3 donors at 10:1 E:T ratio, in the presence of pAXL×CD3ε (2.5 µg/mL). Cells were incubated for 72 h at 37 °C and 5% CO_2_. Cytotoxicity was assessed by flow cytometry monitoring MFI in GFP-positive cells.

For microscope imaging, co-cultured cells were plated on a round cover glass (Fisher Scientific) above 24 wells, and fixed using 4% paraformaldehyde (PFA) for 15 min. Sections were washed three times with PBS 1X, mounted in Vectashield with DAPI (Vector Lab, Newark, CA, USA) and analyzed using Thunder Imaging Systems (Leica, Wetzlar, Germany).

### 2.7. Cell Viability Assay

Cells were plated in 96 wells treated with different concentrations of pAXL×CD3ε, and cell viability was evaluated by Cell Titer-Glo Luminescent Assay (CTG; Promega, Madison, WI, USA), as previously reported [41].

### 2.8. Western Blot

Whole-cell protein extracts were obtained using NP40 lysis buffer containing Halt Protease and Phosphatase Inhibitor cocktail (Invitrogen, Thermo Fisher Scientific), separated using 4–12% Novex Bis-Tris SDS-acrylamide gels (Invitrogen), and transferred on nitrocellulose membranes (Bio-Rad, Hercules, CA, USA), as previously reported [42]. Nitrocellulose membranes were incubated O/N at 4 °C with primary antibody. In detail, anti-AXL (#4566) by Cell Signaling Technology (Danvers, MA, USA) and anti-GAPDH (sc-25778) by Santa Cruz (Dallas, TX, USA) were used for Western blotting (WB) procedures. The membrane was washed thrice with PBS-Tween and incubated with the secondary antibody (anti-rabbit IgG HRP-linked antibody #7074S, Cell Signaling Technology) for 1 h at RT. Chemiluminescence was recorded using SuperSignal West Pico PLUS Chemiluminescent Substrate (Thermo Scientific). Densitometric analysis of blots was performed using LI-COR Image Studio Digits Ver 5.0 (Bad Homburg, Germany).

### 2.9. RNA Isolation and Quantitative Real-Time PCR

The WizPrep™ Total RNA Mini Kit (Wizbiosolutions, Seongnam, South Korea) was used, according to the manufacturer’s guidelines, to extract purified RNA from sarcoma cell lines. The RNA quantity and quality were assessed by NanoDrop^®^ (ND-1000 Spectrophotometer). cDNA was obtained from the reverse transcription of total RNA, using the “high-capacity cDNA reverse transcription kit” (Applied Biosystems, Foster City, CA, USA). Taq-Man^®^ assay (Life Technologies, Carlsbad, CA, USA) was used to detect and quantify AXL (Hs01064439_m1), and GAPDH (Hs03929097_g1) was considered to normalize the recorded threshold cycle values. qRT-PCR was performed in triplicate and relative expression was obtained through the comparative cross threshold method on a ViiA7 System (Thermo Fisher Scientific, Waltham, MA, USA).

### 2.10. T-Cell Activation

Sarcoma cells lines were co-cultured with PBMCs from at least 3 donors at selected 10:1 E:T ratio in the presence of increasing concentrations of pAXL×CD3ε (0.1 µg/mL, 1 µg/mL, and 2.5 µg/mL) or negative control, and were incubated for 72 h at 37 °C and 5% CO_2_. T cells were stained using anti-human CD4 (SK3) FITC (#345768), CD8 (SK1) APC-Cy7 (#641400), CD25 APC (#555434), CD69 PE (#555531), CD3 (UCHT1) PerCP-Cy5.5 (#560835), CD45 (HI30) BV510 (#563204) and CD107a PE (#555801) (BD Biosciences), for 4 h at 37 °C and 5% CO_2_. T cells were selected as CellTrace™ Violet-positive, gated for CD4-, CD8- or CD3-positive, and CD69-, CD25- or CD107a-positive cells. The intracellular production of cytokines and cytolytic enzymes was investigated adding brefeldin A 10 mg/mL. After 4 h, cells were incubated with surface antibodies and treated using FIX&PERM^®^ kit (Nordic MUbio, Susteren, The Netherlands), according to the manufacturer’s guidelines. Subsequently, cells were incubated with anti-TNFα PE-Cy™7 (Mab11) (#560678), anti-IFNγ PE (#559327) and anti-Granzyme B (AlexaFluor^®^647) (#560212) (BD Biosciences) for 15 min at RT in the dark. Samples were finally washed in PBS 1X and analyzed by a flow cytometer.

### 2.11. Analysis of the Activity of pAXL×CD3ε in Combination with Chemotherapeutic Drugs

SAOS-2 were plated in 24 wells and co-cultured at selected 10:1 E:T ratio with PBMCs from at least 3 donors, labelled with CellTrace™ Violet. Cells were treated with pAXL×CD3ε (1 µg/mL), trabectedin (0.2 nM) or their combination (pAXL×CD3ε + trabectedin). After 72 h of incubation, cells were stained using 7-AAD and analysis of positive cells were performed through flow cytometry.

### 2.12. In Vivo Studies

In vivo experiments were performed according to standard guidelines and approved protocols by the National and Institutional Animal Committee (483/2020-PR, 18 May 2020). Four-to-six-week-old male NSG (NOD.Cg-PrkdcscidIl2rgtm1Wjl/SzJ) mice were purchased from Charles River Laboratories (Wilmington, MA, USA). Animals were regularly monitored and euthanized when signs of disease-related symptoms or graft-versus-host disease (GvHD) developed.

To obtain a subcutaneous (sc) xenografted in vivo model, 10 mice were inoculated in the dorsal right flank with HT-1080 cells (3 × 10^6^) resuspended in 100 µL of PBS 1X. On day 4, 10 × 10^6^ PBMCs from healthy donors were intraperitoneally (ip) injected into each mouse. The same day, mice were randomized in 2 groups (5 mice for each group), and 0.1 mg/kg pAXL×CD3ε or vehicle were ip injected for 15 consecutive days. Tumor sizes were measured with a digital caliper. The tumor volume (tv) was calculated using the formula:

tv = (W^2^ × L)/2, (1)

where W is the tumor width and L is the tumor length, as previously described [43].

Mice were sacrificed when the tv reached >2000 mm^3^. At the time of sacrifice, blood samples were collected. Red blood cell lysis was performed, and cells were stained with anti-human CD45 BV510 and CD3 PerCP-Cy5.5 to evaluate PBMCs engraftment. Explanted tumors were analyzed by WB, as previously described, using anti-caspase-3 (#9668, Cell Signaling Technology) and anti-PARP (#9532, Cell Signaling Technology), and by immunohistochemistry (IHC) using anti-CD3 antibody (#GA503, Agilent Dako, Glostrup, Denmark).

### 2.13. Statistical Analysis

Statistical evaluations were carried out using a parametric Student’s *t*-test by the GraphPad software (www.graphpad.com, accessed on 10 February 2023). Graphpad Prism version 6.0 was used to make graphs. Only results with a *p* value < 0.05 were accepted as statistically significant. Each value is reported as the mean of at least 2 experiments ± SD/SEM.

## 3. Results

### 3.1. Evaluation of AXL Expression on Sarcoma Cell Lines

**Figure 1:**
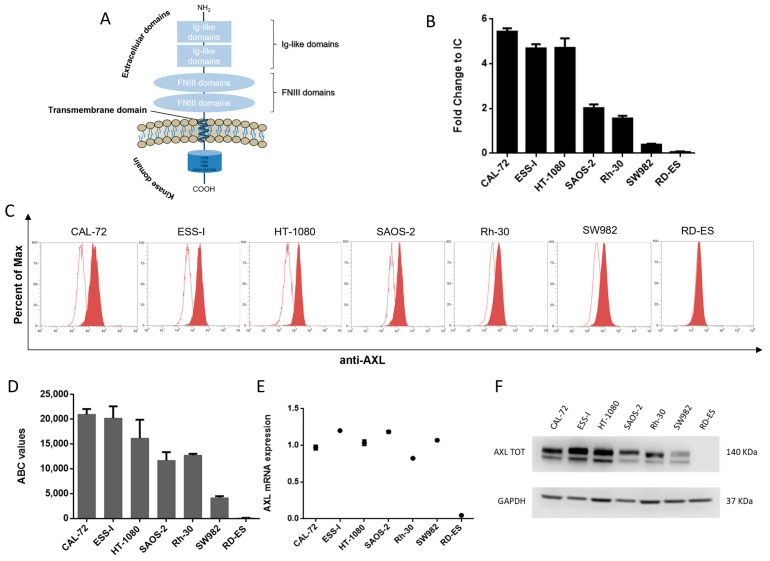
AXL expression. (**A**) Schematic representation of AXL receptor tyrosine kinase structure. It is composed of two immunoglobulin (Ig)-like domains that characterize extracellular domains, two fibronectin type III (FNIII) domains, a transmembrane domain and a kinase domain that is intracellular. (**B**) Flow cytometry analysis of AXL expression of sarcoma cells. (**C**) Representative FACS overlays between unstained (empty) and stained (full red) sample of each cell line. (**D**) Quantification of antibody-binding capacity (ABC) assay. (**E**) AXL-relative mRNA level determined by qRT-PCR and normalized on GAPDH housekeeping. (**F**) Western blot of AXL total form reported in a collection panel of sarcoma cells. The uncropped blots are shown in Figure S3.

To investigate the expression of AXL on sarcoma cells (Figure 1A), we collected a panel of seven human cell lines, including CAL-72 (osteosarcoma), ESS-I (endometrial stromal sarcoma), HT-1080 (fibrosarcoma), SAOS-2 (osteosarcoma), Rh-30 (rhabdomyosarcoma), SW982 (synovial sarcoma) and RD-ES (Ewing’s sarcoma). Flow cytometry showed different AXL expression levels on the surface of tumor cells: high expression on CAL-72, ESS-I and HT-1080; intermediate expression on SAOS-2 and Rh-30; and low- or no-expression on SW982 and RD-ES cell lines, respectively (Figure 1B,C). This trend was confirmed performing quantitative analysis of antigen expression density for each cell line, using calibrated microspheres to assess the antibody-binding capacity (ABC). As reported in Figure 1D, AXL expression was in a range between 21,000 and 4200 antigen molecules on CAL-72 and SW982 cells, respectively. Through qRT-PCR analysis, we assessed the AXL mRNA expression in sarcoma cells (Figure 1E). Our findings revealed a different pattern of target expression, which was in accordance with data retrievable by cBioPortal for the Cancer Genomics dataset (cbioportal.org, accessed on 10 February 2023) and Cancer Cell Line Encyclopedia (CCLE) dataset (https://depmap.org/portal/interactive, accessed on 10 February 2023). Further Western blot analyses were performed to investigate the expression of AXL protein in sarcoma cells, reporting a clear difference in the band intensity between various cell lines (Figure 1F). According to our results, AXL expression is not correlated to specific sarcoma sub-types.

Our data demonstrate that AXL is highly expressed in sarcoma cells, therefore representing a potential mean for selective targeting. The interaction of pAXL×CD3ε with sarcoma cells was assessed through indirect staining, using an anti-human IgG secondary antibody (Jackson ImmunoResearch, West Grove, PA, USA) (Figure S1).

### 3.2. T-Cell Mediated Cytotoxicity Is Induced by pAXL×CD3ε In Vitro

**Figure 2:**
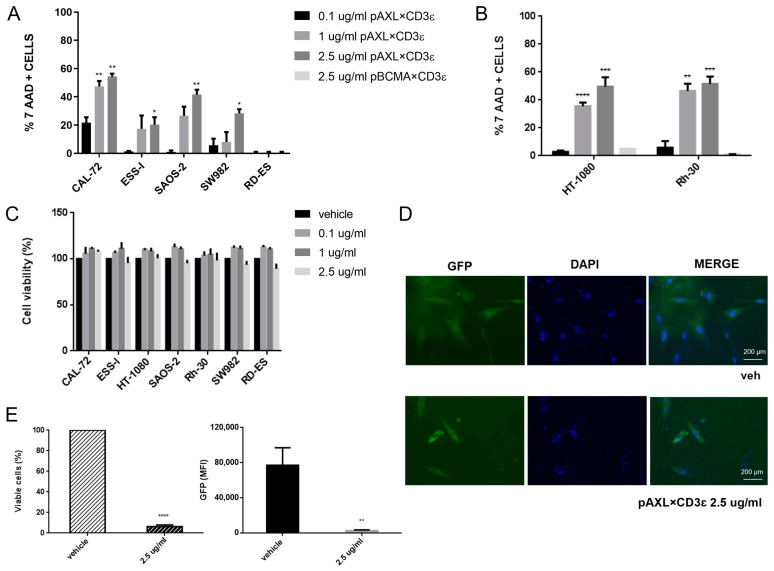
Redirected T-lymphocyte cytotoxicity by pAXL×CD3ε in sarcoma cell lines. (**A**) FACS analysis of 7-AAD(7-amino-actinomycin D)-positive cells. (**B**) HT-1080 and Rh-30 treated with increasing concentrations of pAXL×CD3ε (0.1 µg/mL, 1 µg/mL and 2.5 µg/mL), and as a negative control 2.5 µg/mL of pBCMA×CD3ε after 72 h. Each group has been compared to the 0.1 µg/mL group for statistical analysis. Results are normalized to the recorded vehicle values. (**C**) Cell Titer-Glo luminescent cell viability (%) assay performed on sarcoma cell lines without effector cells, following pAXL×CD3ε 72 h treatment. (**C**) Positive cells (relative percentage %) of sarcoma cell lines co-cultured with peripheral blood mononuclear cells (PBMCs) and treated with different concentrations of pAXL×CD3ε or vehicle for 72 h. Each group has been compared to the 0.1 µg/mL group for statistical analysis. Results are normalized to the recorded vehicle values. (**D**) Imaging of CAL-72 stably expressing green fluorescent protein (GFP) in untreated cells (vehicle) and 2.5 µg/mL of pAXL×CD3ε-treated cells after 72 h. Nuclei were stained with DAPI and microscopies were performed at 10-fold magnification. (**E**) Percentage of stably expressing CAL-72 GFP viable cells and median fluorescence intensity (MFI) of GFP analyzed by flow cytometry. PBMCs were obtained from 3 healthy donors and results are expressed as the mean value of triplicate experiments from each donor. * *p* < 0.0332; ** *p* < 0.0021; *** *p* < 0.0002; **** *p* < 0.0001.

To assess the activity of pAXL×CD3ε, sarcoma cells with different expression levels of AXL were co-cultured with purified human T cells (E:T ratio selected at 10:1) from healthy donors, in the presence of three different concentrations of pAXL×CD3ε (0.1 µg/mL, 1 µg/mL, and 2.5 µg/mL) for 72 h. Increasing concentrations of pAXL×CD3ε produced T-cell-mediated cytotoxicity on sarcoma cells except for the RD-ES because of its low expression of the target antigen (Figure 2A,B). In particular, the cytotoxic activity of pAXL×CD3ε followed a binding-response effect leading to around 50% of cell death on CAL-72, Rh-30, and HT-1080, 40% on SAOS-2, 30% and 20% in SW982 and ESS-I, respectively, at 2.5 µg/mL. As a negative control, we performed a cytotoxicity assay using a Pronectin™-based BTCE binding a different target expressed by B cells only, the BCMA, not expressed by sarcoma cells (pBCMA×CD3ε).

As shown in Figure 2B, 2.5 µg/mL of pBCMA×CD3ε did not induce cytotoxicity in two different sarcoma cell lines, such as HT-1080 and Rh-30, when it was used at a higher dose. To exclude direct cytotoxicity of pAXL×CD3ε on sarcoma cells, we performed a cell viability assay that allows the evaluation of metabolically active cells, in the absence of effector cells. We found that different concentrations of pAXL×CD3ε did not alter cell growth capability of cancer cells (Figure 2C), indicating that T lymphocytes are indeed required to induce the redirected cytotoxicity of sarcoma cell lines. Furthermore, we performed co-culture experiments on sarcoma cells stably expressing GFP in the presence of 2.5 µg/mL of pAXL×CD3ε. As expected, we observed a strong reduction of GFP signal via imaging analysis, confirming the cytotoxic activity of pAXL×CD3ε observed in our experimental models (Figure 2D and Figure S2A). Moreover, the percentage of viable cells and fluorescence quantification evaluated by flow cytometry led to the same result (Figure 2E and Figure S2B).

Taken together, our findings demonstrate that pAXL×CD3ε has an antitumor effect through the recruitment of cytotoxic T lymphocytes.

### 3.3. pAXL×CD3ε Triggers T-Lymphocyte Activation against Sarcoma Cells

**Figure 3:**
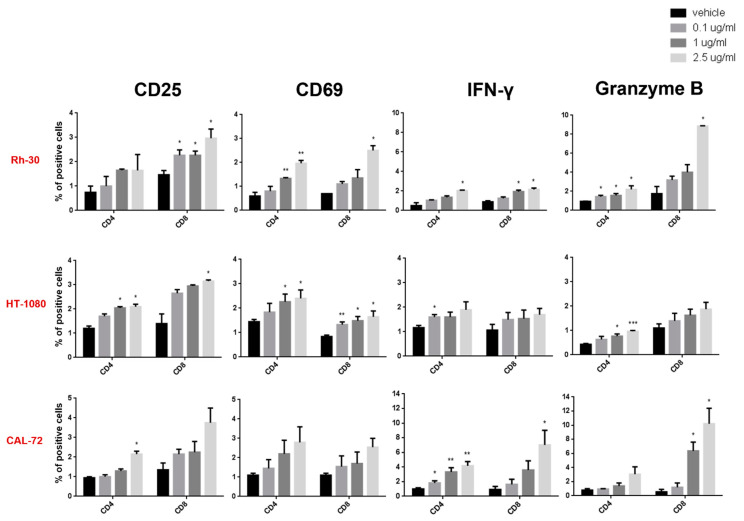
Functional experiments on CD4–CD8 gated T cells. Surface early and late activation markers (CD69 and CD25), cytokine release (IFN-γ) and cytolytic enzyme (Granzyme B) on CD4 and CD8-positive T lymphocytes from at least 3 donors co-cultured with Rh-30, HT-1080 and CAL-72 sarcoma cell lines at 10:1 E:T ratio, in the presence of different concentrations of pAXL×CD3ε. Each result is expressed as the mean value of triplicate experiments obtained from each donor. * *p* < 0.0332; ** *p* < 0.0021; *** *p* < 0.0002.

Functional effects on PBMCs co-cultured with sarcoma cells at 10:1 E:T ratio, in the presence of increasing concentrations of pAXL×CD3ε or vehicle, were also evaluated after 72 h of treatment. As shown in Figure 3, we observed the upregulation of early and late T-cell surface activation markers (CD69 and CD25) in experiments performed in three different sarcoma cell lines, such as Rh-30, HT-1080 and CAL-72. Additionally, pAXL×CD3ε induced the release of inflammatory cytokine Interferon-γ (IFN-γ) and cytolytic enzyme, Granzyme B.

**Figure 4:**
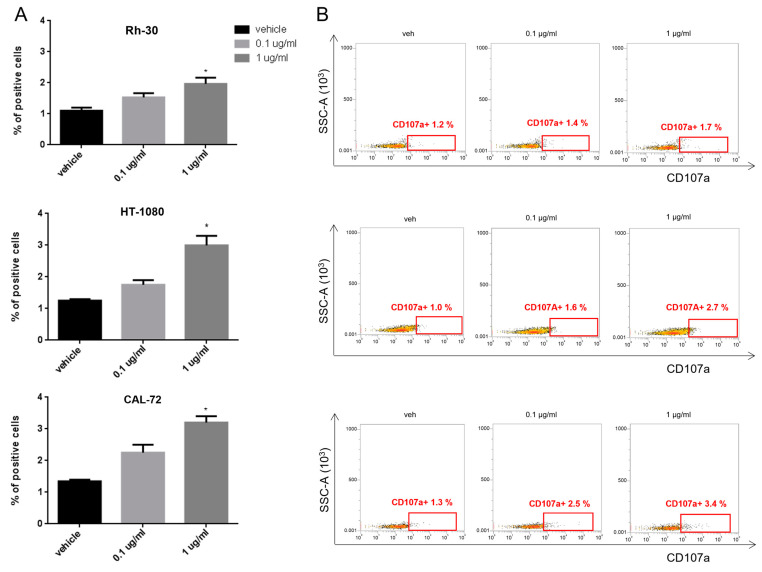
Degranulation assay on activated T lymphocytes. (**A**) Histogram quantification of CD107a-positive T cells from at least 3 donors, co-cultured with Rh-30, HT-1080 and CAL-72 sarcoma cell lines and treated with 0.1 µg/mL and 1 µg/mL of pAXL×CD3ε for 72 h. (**B**) Representative dot plots of CD107a-positive T lymphocytes analyzed by flow cytometry. Each result is expressed as the mean value of triplicate experiments obtained from each donor. * *p* < 0.0332.

Consistent with their cytotoxic function, T lymphocytes were also positive for CD107a degranulation marker (Figure 4A,B).

These data indicate that pAXL×CD3ε produces a dose-dependent activation of T lymphocytes against AXL-positive sarcoma cells.

### 3.4. pAXL×CD3ε Increases Cytotoxicity Induced by Trabectedin

**Figure 5:**
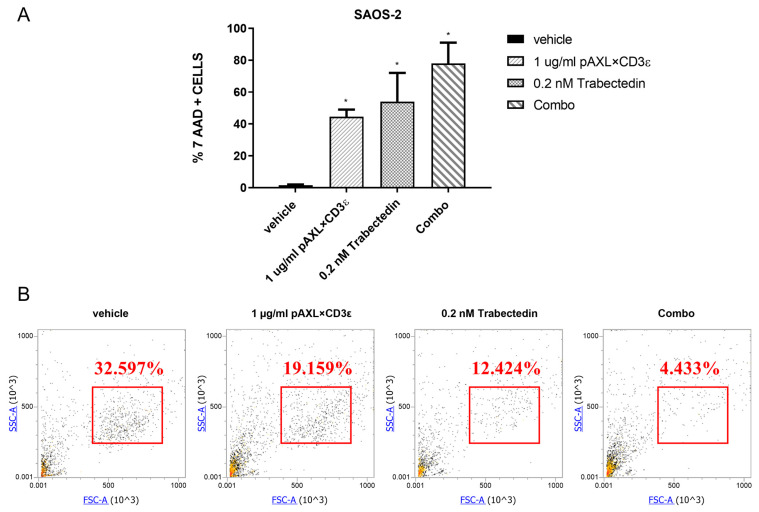
pAXL×CD3ε sensitizes chemotherapeutic drug activity in vitro. (**A**) Redirected cytotoxicity T-cell of pAXL×CD3ε in SAOS-2, analyzed in co-culture experiments using 1 µg/mL of pAXL×CD3ε, 0.2 nM of trabectedin and their combination (Combo) for 72 h. (**B**) Representative dot plots of SAOS-2 viability reduction (%) after treatment with pAXL×CD3ε and trabectedin alone or in combination. PBMCs were obtained from 3 healthy donors and results are expressed as the mean value of triplicate experiments from each donor. * *p* < 0.0332.

To verify if pAXL×CD3ε could make tumor cells more sensitive to conventional chemotherapeutic drugs, SAOS-2 was selected as the cell model to investigate the effect of redirected T-cell toxicity. Cells were co-treated with pAXL×CD3ε (1 µg/mL) and trabectedin (0.2 nM). After 72 h of treatment, an enhanced cytotoxic effect was observed for pAXL×CD3ε plus trabectedin, as compared to the effect induced by the single agents. In detail, pAXL×CD3ε increased cell death >20% in SAOS-2 cells compared to the effect induced by trabectedin alone (Figure 5A). The dot plots in Figure 5B provide a graphical overview of the reduction in cell viability (%). These data suggest a potential advantage induced by the combination of pAXL×CD3ε with chemotherapeutics commonly used for sarcoma therapy.

### 3.5. pAXL×CD3ε In Vivo Activity

**Figure 6:**
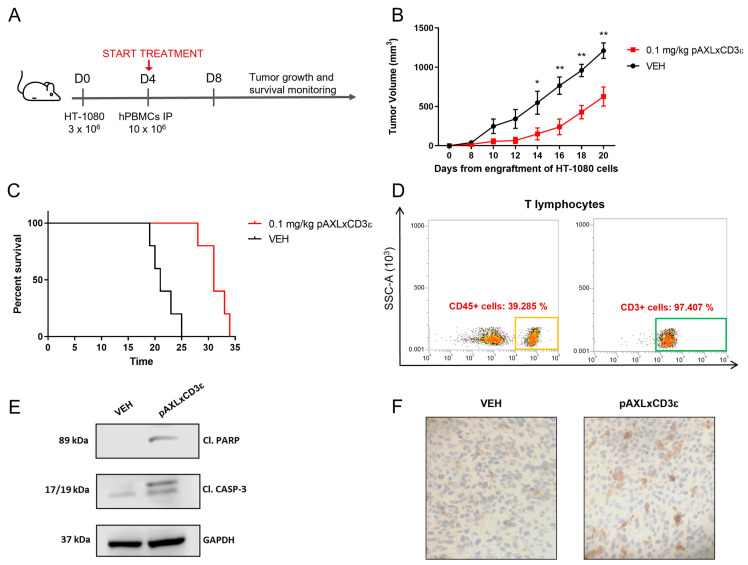
pAXL×CD3ε in vivo activity. (**A**) Experimental timeline of in vivo study on a sarcoma xenograft model. (**B**) Tumor volume curve of mice treated with pAXL×CD3ε 0.1 mg/kg or vehicle alone. Results obtained from each group have been compared at the same timepoint for statistical purposes. (**C**) Survival curves (Kaplan–Meier) of mice treated with pAXL×CD3ε or vehicle. (**D**) Representative FACS dot plots of T-cell engraftment evaluated on the day of sacrifice on intracardiac blood samples collected from mice. CD3-positive cells were evaluated on gated CD45-positive lymphocytes. (**E**) WB analysis of cleaved caspase-3 and cleaved PARP in whole-cell protein extracts from representative retrieved xenografts. GAPDH was used as a loading control. The uncropped blots are shown in Figure S3. (**F**) IHC staining of CD3 lymphocytes performed on tumors explanted from mice treated with vehicle or pAXL×CD3ε 0.1 mg/kg at 20-fold magnification. * *p* < 0.0332; ** *p* < 0.0021.

The in vivo antitumor efficacy of pAXL×CD3ε was validated against human HT-1080 cell xenografts in NSG-immunocompromised mice (Figure 6A). A total of 10 xenografted mice were randomized to receive pAXL×CD3ε (0.1 mg/kg, five mice) or the vehicle alone (VEH, five mice) as the control group. A significant reduction of tumor growth was observed in NSG mice treated with pAXL×CD3ε as compared to VEH (Figure 6B). After 20 days from the cell engraftment, mice treated with pAXL×CD3ε showed a tumor volume of about 630 mm^3^ versus 1200 mm^3^ in the VEH-only group. This effect translated into a prolonged survival of treated animals (Figure 6C). To demonstrate the engraftment of human T-lymphocytes in these immunocompromised mice, flow cytometry analyses were performed on peripheral blood samples collected from mice on the day of sacrifice. An anti-CD3-fluorochrome-conjugated antibody was used for the staining, and T-cell engraftment was confirmed both in pAXL×CD3ε and VEH groups (Figure 6D). Retrieved xenografts from mice were homogenized and WB analysis was performed on the whole-cell protein extracts. The analysis revealed the induction of apoptotic processes, which was demonstrated by the increase of cleaved PARP and cleaved caspase-3 in treated mice as compared to VEH (Figure 6E). IHC analyses also highlighted the infiltration of CD3+ cells in tumor xenografts from mice treated with pAXL×CD3ε, thus demonstrating the effective engagement of T-lymphocytes at the tumor site (Figure 6F).

Based on these findings, pAXL×CD3ε demonstrates promising antitumor activity against sarcoma xenografts in vivo.

## 4. Discussion

Cancer immunotherapy based on T-cell engagement is a valuable therapeutical option and is in an advanced phase of clinical evaluation for different hematological malignancies [44,45,46]. While conventional mAbs bind the same antigen with both fragment antigen-binding (Fab) arms [47], BTCEs simultaneously bind a TAA on cancer cells and the epsilon (ε) subunit of CD3 on the T lymphocytes and, therefore, can efficiently trigger redirected T-cell cytotoxicity in an MHC-independent fashion [48,49]. This simultaneous engagement of the antigen on tumor cells and effector cells leads to an immunological synapse, resulting in T-cell activation and subsequent release of inflammatory cytokines and cytolytic molecules that lead to the killing of cancer cells [39,40,50].

Despite their demonstrated efficacy in patients with hematological malignancies, no BTCEs have been approved so far for the treatment of solid tumors [51]. There are, in fact, some main hurdles that can hamper the use of BTCEs in solid tumors: (i) on-target off-tumor toxicities due to the absence of specific TAAs; (ii) impaired anti-cancer activity due to the hostile and immunosuppressive tumor microenvironment (TME) that antagonizes T-cell infiltration into the tumor mass; (iii) reduced bioavailability and scarce penetration within a solid tumor mass [52,53]. In this scenario, the TME may play a relevant role in cancer progression and can influence the clinical management of these diseases [54,55,56], since it includes immune cells and stromal cells interacting with malignant cells through contact mechanisms or cytokines and subcellular structures, inducing both pro-tumor and antitumor activity. Among them, CD4+ and CD8+ T cells, together with NK cells, dendritic cells, and M1 tumor-associated macrophages (TAMs), promotes cell killing [57,58,59]. Novel approaches would aim to restore the immune function overcoming cancer suppressive effects [60]. In this light, different strategies are emerging by innovative protein-based scaffolds and novel targets [39,40,61].

Here, we assessed that AXL is highly expressed among a variety of sarcomas, confirming previous data performed on primary sarcoma samples [20,22,31,32,62,63] and representing a promising target for the development of innovative immunotherapeutic approaches, especially in chemo-refractory disease. Previous studies, using mAbs against AXL, have reported activity and manageable toxicity in sarcoma patients, suggesting its targeting potential also in the clinical setting [22,64]. Recently, different strategies based on mAbs and CAR-T cells showed encouraging results against AXL-expressing sarcomas and some of them are currently under clinical investigation. The safety and tolerability of CCT301-38 CAR-modified autologous T cells are being investigated in subjects with r/r sarcomas (NCT05128786). Patients with AXL gene alterations were also recruited for another phase I study to determine the safety, tolerability, pharmacokinetics, and anti-tumor effects induced by Mipasetamab Uzoptirine (ADCT-601) alone, or in combination with other anti-cancer drugs (NCT05389462). The immunogenicity and antitumor efficacy of BA3011, a conditionally active biologic (CAB) AXL-targeted antibody drug conjugate (CAB-AXL-ADC), is being investigated in a phase I/II study in different sarcoma subtypes, in monotherapy or combined with a PD-1 inhibitor (NCT03425279). Finally, a trial has been completed on different tumor types, including sarcoma, to investigate the safety and efficacy of Enapotamab Vedotin (HuMax-AXL-ADC), an AXL-specific antibody drug conjugate (NCT02988817).

On these premises, we focused on AXL as an immunotherapeutic target to be exploited in sarcoma treatment by BTCE-based strategy. We used an emerging protein therapeutic class, called Pronectins™ [36], taking advantage of their small size and low molecular weight to reach a higher concentration within the tumor tissue. Consistently, here we demonstrated that pAXL×CD3ε indeed redirects T cells toward AXL-expressing sarcoma cells, leading to a dose-dependent T-cell activation, with a consequent release of inflammatory cytokines and cytolytic molecules. Our results are in accordance with data recently reported, showing that pAXL×CD3ε exhibits cytotoxic effects on AXL-positive MDA-MB-231 cells and minimal cytotoxicity on AXL-negative CHO cells [38]. Moreover, we found enhanced cytotoxic effects as a function of increased concentrations of pAXL×CD3ε, which was highly promising taking into account the presence of immune cells in the TME of sarcomas. Furthermore, we demonstrated that the combination of pAXL×CD3ε with trabectedin, a conventional active chemotherapeutic drug, improved the cytotoxicity of sarcoma cells. Even if the use of conventional chemotherapeutics is under interindividual variability term of efficacy and toxicity [65,66,67], our data suggest the feasibility of combinatorial treatments and are consistent with preliminary reports, which showed anti-sarcoma activity of immunotherapy/chemotherapy combination [68]. Importantly, in our in vivo model, pAXL×CD3ε guaranteed the recruitment of T lymphocytes in the tumor site and significantly inhibited the growth of sarcoma xenografts, suggesting that this strategy has the potential to also control the fast-growing tumor cells in patients. These findings are of translational relevance, since conventional approaches are still largely unsuccessful, and the only favorable strategy at the present, for the treatment of this incurable disease, is represented by surgery in combination with pre- or post-surgery therapies.

Overall, we demonstrate that the first-in-class pAXL×CD3ε-based immunotherapy exerts significant anti-sarcoma activity in vitro and in vivo, and therefore represents a promising tool to be developed in the clinical setting, offering a novel opportunity to overcome the unmet need of long-term control of drug refractory disease.

## 5. Conclusions

Despite the identification of molecular mechanisms driving sarcoma genesis, as well as the discovery of key transcription factors, sarcoma treatment still represents a great challenge. The variability in response to current therapies can be ascribed to their heterogeneity and aggressive behavior. Taken together, our results indicate that AXL-targeting by the Pronectin™-based BTCE platform may represent a new-generation strategy for the treatment of this still-incurable disease.

## Data Availability

The data presented in this study are available in this article and Supplementary Materials.

## Supplementary Materials

The following supporting information can be downloaded at: https://www.mdpi.com/article/10.3390/cancers15061647/s1, Figure S1: Interaction of pAXL×CD3ε with sarcoma cells; Figure S2: Redirected T-lymphocyte cytotoxicity by pAXL×CD3ε in sarcoma cell lines; Figure S3: Original Western blots.
