# Supplementary material for: The First-In-Class Anti-AXL×CD3ε Pronectin™-Based Bispecific T-Cell Engager Is Active in Preclinical Models of Human Soft Tissue and Bone Sarcomas
Source: Cancers (Basel). 2023 Mar 8;15(6):1647. doi: 10.3390/cancers15061647 (PMC10046451; doi:10.3390/cancers15061647)
Supplement: Supplementary file 1 [file cancers-15-01647-s001.zip › cancers-2249798-supplementary.pdf]

cancers

MDPI

Supplementary Materials

The First-In-Class Anti-AXL×CD3ε Pronectin™-Based Bispecific T-Cell Engager Is Active in Preclinical Models of Human Soft Tissue and Bone Sarcomas

Nicoletta Polerà, Antonia Mancuso, Caterina Riillo, Daniele Caracciolo, Stefania Signorelli, Katia Grillone, Serena Ascrizzi, Craig A. Hokanson, Francesco Conforti, Nicoletta Staropoli, Luigia Gervasi, Maria Teresa Di Martino, Mariamena Arbitrio, Giuseppe Nisticò, Roberto Crea, Pierosandro Tagliaferri, Giada Juli and Pierfrancesco Tassone

Interaction of pAXL×CD3ε with sarcoma cells

To confirm the interaction of pAXL×CD3ε, sarcoma cell lines were stained with 1 µg/ml of human IgG or pAXL×CD3ε for 15 min at room temperature in the dark. Then, samples were washed in PBS 1X and incubated with an anti-human IgG AlexaFluor®647 secondary antibody (Jackson ImmunoResearch). The tubes were washed in PBS 1X, resuspended in 500 µL and analysed by flow cytometry.

Redirected T-cell cytotoxicity assay

Sarcoma cells stably expressing green fluorescent protein (GFP) were co-cultured with PBMC at 10:1 E:T ratio, in presence of 2.5 µg/ml pAXL×CD3ε. Cells were incubated for 72 h at 37°C and 5% CO_2. Cytotoxicity was assessed by flow cytometry monitoring MFI in GFP positive cells.

For microscope image acquisition, co-cultured cells were plated on round cover glass (Fisher Scientific) above 24 well and fixed using 4% paraformaldehyde (PFA) for 15 min. Sections were washed three times with PBS 1X, mounted in Vectashield with DAPI (Vector Lab) and analyzed using Thunder Imaging Systems (Leica).

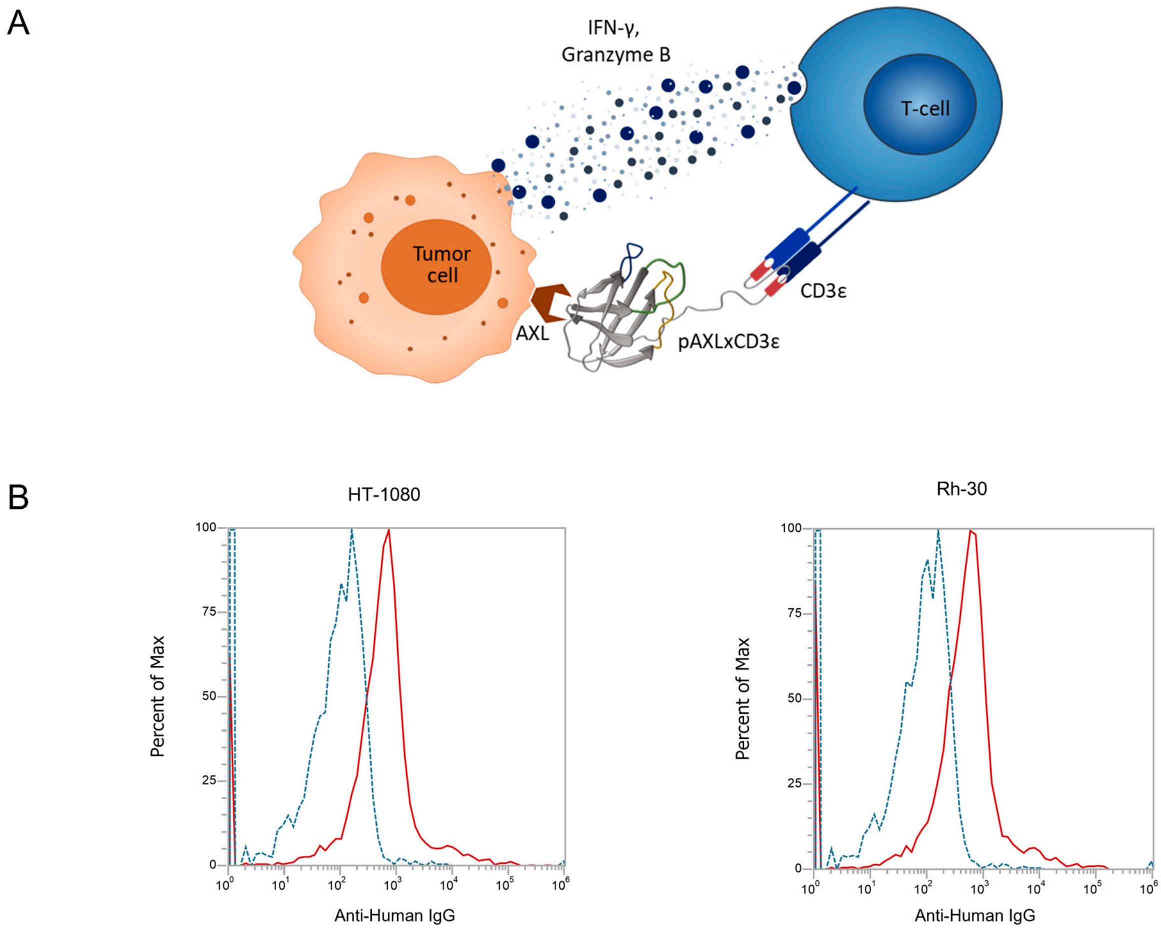

Figure S1. Interaction of pAXL×CD3ε with sarcoma cells. A) Schematic representation of the mechanism of action of pAXL×CD3ε redirecting T cells against tumor cells. B) Representative FACS overlays between Human IgG1 Isotype Control with secondary antibody (blue) or pAXL×CD3ε with secondary antibody (red) in two different AXL expressing sarcoma cell lines. All antibodies were used at a final concentration of 1 μg/ml.

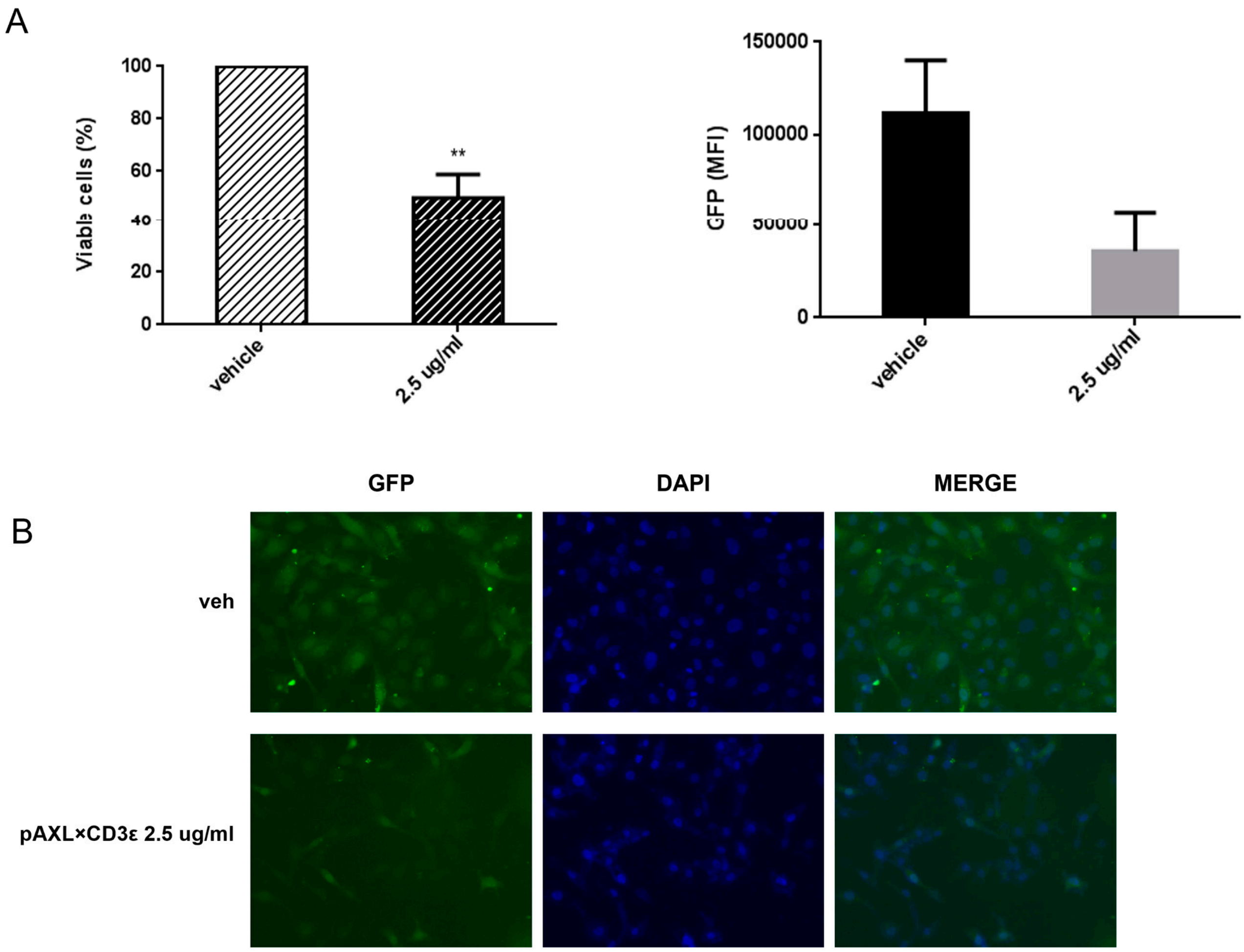

Figure S2. Redirected T-lymphocytes cytotoxicity by pAXL×CD3ε in sarcoma cell lines. A) Percentage of stably expressing HT-1080 GFP viable cells and median fluorescence intensity (MFI) of GFP analyzed by flow cytometry. B) Imaging of HT-1080 stably expressing green fluorescent protein (GFP) in untreated cells (vehicle) and 2.5 μg/ml of pAXL×CD3ε treated cells after 72 h. Nuclei were stained with DAPI and microscopies are performed at 10-fold magnification. PBMCs were obtained from 3 healthy donors and results are expressed as the mean value of triplicate experiments obtained from each donor. **p<0.0021.

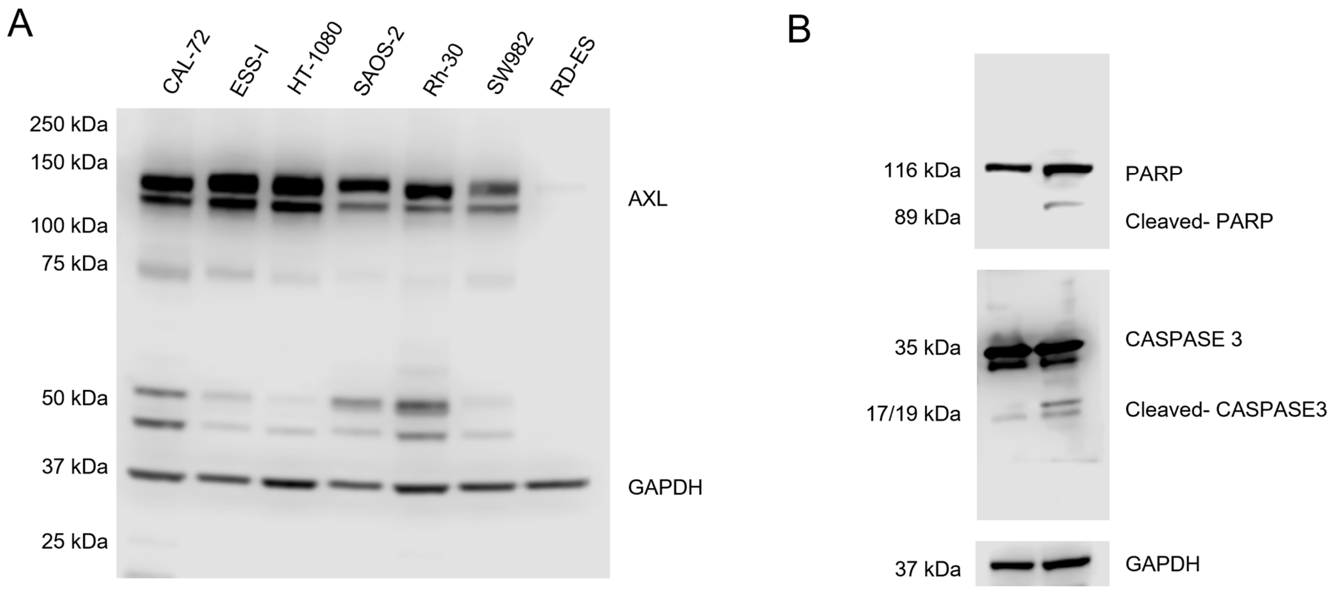

Figure S3. Original western blots. The whole Western blot referred to Figure 1F (A) and Figure 6E (B) showing all bands and molecular weight markers are reported.
